# External Validation and Clinical Impact of the Barcelona Predictive Models for Detecting Significant Prostate Cancer in Prostate Biopsies in an Ibero-American Population

**DOI:** 10.3390/cancers18111810

**Published:** 2026-06-01

**Authors:** Nahuel Paesano, Juan Camean, Maximiliano Ringa, Maximiliano López-Silva, Guido Koren, Tomás Eduardo Olmedo, Joaquín Ignacio Gurovich, Edgar Iván Bravo-Castro, Violeta Catalá, Pablo Contreras, Juan Justo-Quintas, José Miguel Pérez-Ruiz, Silvia García-Barreras, Berta Miró, Lucas Regis, Olga Méndez, Enrique Trilla, Juan Morote

**Affiliations:** 1 Department of Urology, ROC Clinic and HM Hospitals, 28050 Madrid, Spain; nahuel.paesano@rocclinic.com (N.P.); jjustoquintas@rocclinic.com (J.J.-Q.); jose.perez@rocclinic.com (J.M.P.-R.); silvia.garcia@rocclinic.com (S.G.-B.); 2 Uro-Radiology Unit, Clinica Creu Blanca, 08034 Barcelona, Spain; violetacatala@uroima.com; 3 Department of Surgical Oncology, Instituto Alexander Fleming, Buenos Aires 1426, Argentina; juanjcamean@gmail.com; 4 Department of Urology, Hospital Alemán, Buenos Aires 1424, Argentina; mringa@hospitalaleman.com (M.R.); pablocontreras.ar@gmail.com (P.C.); 5 Centro Argentino de Urología, Buenos Aires 1114, Argentina; maximilianolopezsilva@gmail.com; 6 Centro de Estudios Médicos e Investigación Clínica, Buenos Aires 1431, Argentina; gkoren@cemic.edu.ar; 7 Department of Urology, Hospital Clínico Universidad de Chile, Independencia, Santiago 8380456, Chile; tolmedo67@gmail.com (T.E.O.); joaquingurovich@gmail.com (J.I.G.); 8 Department of Urology, Hospital Central Militar, Mexico 11200, Mexico; briv_edca@hotmail.com; 9 Bioinformatics and Statistical Unit, Vall d’Hebron Research Institute, 08035 Barcelona, Spain; berta.miro@vhir.org; 10 Department of Urology, Hospital Universitario Vall d’Hebron, 08035 Barcelona, Spain; lucas.regis@vallhebron.cat (L.R.); enrique.trilla@vallhebron.cat (E.T.); 11 Research Group in Urology, Vall d’Hebron Research Institute, 08035 Barcelona, Spain; 12 Department of Surgery, Universitat Autònoma de Barcelona, 08193 Bellaterra, Spain

**Keywords:** prostate cancer, screening, predictive models

## Abstract

**Objectives:** To externally validate the Barcelona Predictive Models (BCN-PM 1 and 2) for detecting csPCa in an Ibero-American population. BCN-PM 1 was designed to reduce magnetic resonance imaging (MRI) use, whereas BCN-PM 2 aims to decrease unnecessary prostate biopsies. **Methods:** This prospective, multicenter study included 661 men with suspected PCa recruited in 2025 across three Ibero-American centers. All participants underwent MRI followed by targeted biopsies of lesions with the Prostate Imaging–Reporting and Data System (PI-RADS) ≥ 3, along with systematic biopsy. When PI-RADS lesions were <3, only systematic biopsies were performed. CsPCa was defined as International Society of Urological Pathology Grade Group ≥ 2. BCN-PM 1 incorporates age (years), family history of PCa (no vs. yes), prior negative prostate biopsy (no vs. yes), digital rectal examination (DRE: normal vs. suspicious), and prostate volume-derived estimation by DRE (small, median, or large). BCN-PM 2 includes age, family history of PCa, prior negative prostate biopsy, prostate volume measured by MRI (mL), and PI-RADS score (1–5). **Results:** The rate of csPCa detection was 53.7%. Both models demonstrated good calibration with strong agreement between predicted probabilities and observed csPCa rates. BCN-PM 1 closely followed the reference line, with minor deviations at higher predicted probabilities, whereas BCN-PM 2 showed modest departures at the extremes of risk. The area under the curve was 0.740 (95% CI 0.702–0.777) for BCN-PM 1 and 0.803 (95% CI 0.769–0.836) for BCN-PM 2 (*p* < 0.001). Decision curve analysis demonstrated a net benefit for both models compared with strategies of biopsy in all or no men. BCN-PM 2 showed greater net benefit than BCN-PM 1. At 95% sensitivity, BCN-PM 1 reduced MRI requests by 10.6%, while BCN-PM 2 avoided 19.4% of unnecessary biopsies. The sequential use of BCN-PM 1 and 2 resulted in a 10.6% reduction in MRI exams and a 23.1% reduction in biopsies, at the cost of missing 8.4% of csPCa cases. The performance of the biopsy improved from 53.7% to 64.0% (*p* < 0.001). **Conclusions:** BCN-PM1 and BCN-PM 2 were successfully validated in an Ibero-American population.

## Simple Summary

Screening for prostate cancer (PCa) reduces disease-specific mortality by enabling the early detection and treatment of clinically significant PCa (csPCa). Suspicion of PCa is typically based on serum prostate-specific antigen (PSA) levels, while magnetic resonance imaging (MRI) is used to estimate the likelihood of csPCa and to guide targeted biopsies of suspicious lesions. Despite these advantages, high rates of unnecessary prostate biopsies and overdiagnosis of insignificant PCa (iPCa) persist. Predictive models can improve the efficiency of csPCa early detection; however, they require validation after being implemented in new populations.

## 1. Introduction

Prostate cancer (PCa) is the most commonly diagnosed malignancy in men worldwide and a leading cause of cancer-related morbidity and mortality [1]. Early detection of clinically significant PCa (csPCa) is essential to reduce disease-specific mortality while minimizing overdetection of insignificant PCa (iPCa) [2,3]. In September 2022, the European Union recommended population-based screening across its Member States, reflecting the growing recognition of the importance of early detection [4,5].

PCa screening has traditionally relied on serum prostate-specific antigen (PSA) measurement, which lacks specificity and often leads to unnecessary biopsies. Pre-biopsy magnetic resonance imaging (MRI) has emerged as a powerful risk-stratification tool, u-sing the Prostate Imaging–Reporting and Data System (PI-RADS) to estimate the probability of csPCa [6,7]. The history of PCa screening illustrates the challenge of balancing early detection with harm avoidance. In 2011, the US Preventive Services Task Force recommended against PSA-based screening due to high rates of unnecessary biopsies, overdetection and overtreatment of iPCa, and treatment-related complications [8]. Rising rates of advanced and metastatic PCa prompted a 2018 revision endorsing shared decision-making after counseling men on overdiagnosis and biopsy-related risks [9]. Active surveillance, MRI-targeted biopsies, and transperineal approaches have improved the management of iPCa and the early detection of csPCa while reducing infectious complications [10]. Nevertheless, unnecessary prostate biopsies and iPCa detection remain frequent, particularly in intermediate-risk scenarios such as PI-RADS 3 [11].

Predictive models (PMs) are tools developed to assess the individual risk of a defined event. Their practical application depends on the availability of user-friendly, free, and rapid risk calculators, as well as external validations prior to implementation in populations different from those used for their development [12]. To optimize csPCa screening, the European Association of Urology (EAU) currently recommends the design of risk-stratified pathways, using predictive models, to reduce MRI requests and select candidates for prostate biopsy [13].

The Barcelona PMs (BCN-PMs) were developed in 2022 to predict csPCa in prostate biopsies before and after MRI. BCN-PM 1 aims to reduce MRI requests, while BCN-PM 2 aims to decrease unnecessary prostate biopsies. Both models use age (years), serum PSA (ng/mL), digital rectal examination (DRE: normal vs. suspicious), family history of PCa (no vs. yes), and prior negative prostate biopsy (no vs. yes) as predictive variables. Additionally, BCN-PM 1 uses an estimation of prostate volume from DRE (small, median, or large) [14], while BCN-PM 2 includes MRI-derived prostate volume (mL) and the PI-RADS score v 2.0 [15]. A free smartphone-based risk calculator is available for iOS and Android (http://bcnrc.com). Subsequently, BCN-PM 2 has been validated in men receiving 5-alpha reductase inhibitors (5-ARI), which were initially excluded in its development [16]. BCN-PM 2 has also been validated in a population using PI-RADS v 2.1, and undergoing transperineal biopsies, which are currently recommended to diagnose csPCa [17]. Finally, BCN-PM 2 has been validated across three European countries [18].

We hypothesized that external validation of BCN-PM 1 and 2 in an Ibero-American population would demonstrate their clinical utility for csPCa detection in prostate biopsies. The main objective was to validate the BCN-PM 1 and 2. Secondary objectives were: (i) to assess model calibration; (ii) to evaluate clinical utility by analyzing discrimination for csPCa, net benefit compared with biopsy-all and biopsy-none strategies, and differences in biopsy avoidance and missed csPCa across risk thresholds; and (iii) to identify the thresholds associated with the clinically relevant 95% sensitivity of and the corresponding rates of biopsy avoidance.

## 2. Materials and Methods

### 2.1. Design, Setting, and Participants

**Figure 1:**
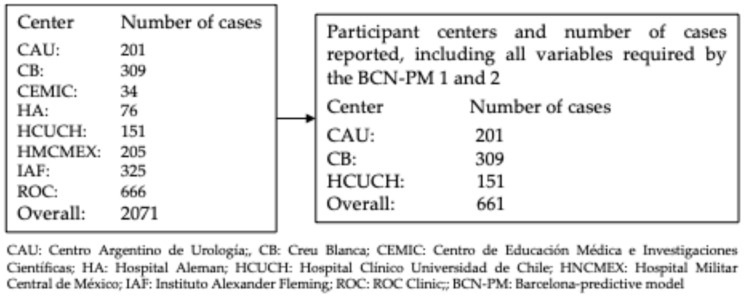
Flowchart of cases reported by participating centers and the final number of cases selected for head-to-head validation of BCN-PM 1 and 2.

A prospective, multicenter study evaluating the opportunistic early detection of PCa in an Ibero-American population in 2025 included 2017 men who underwent MRI followed by targeted and/or systematic biopsies. Eight academic centers—located in Spain (n = 2), Argentina (n = 4), Chile (n = 1), and Mexico (n = 1)—contributed to case recruitment. From the overall cohort, 661 men from CAU (Buenos Aires, Argentina), CB (Barcelona, Spain), and HCUCH (Santiago, Chile) were selected based on the availability of all variables required for a head-to-head validation of the BCN-PM 1 and 2 (Figure 1).

This project was approved by the ethics committee of the coordinating center, Vall d’Hebrón Research Institute (PRAG02/2020), with participants signing informed consent for prostate biopsy and data management.

### 2.2. PCa Suspicion and Diagnostic Procedure of csPCa

PCa suspicion was defined as a serum PSA level > 3.0 ng/mL and/or a suspicious DRE [10]. All men underwent multiparametric MRI on 1.5 or 3 Tesla scanners using a surface phased-array coil, following the recommendations of the European Society of Urogenital Radiology, including T2-weighted, diffusion-weighted, and dynamic contrast-enhanced sequences [19]. MRI examinations were interpreted locally by experienced radiologists using PI-RADS v 2.1 [20,21].

Almost all prostate biopsies were performed via transperineal approach using either cognitive or software-assisted MRI–transrectal ultrasound (TRUS) fusion. For each suspicious lesion (PI-RADS ≥ 3), at least 2–4 targeted cores were obtained (up to three lesions per case), followed by 10–12-core systematic biopsy. Men with PI-RADS < 3 lesions underwent 10–12-core systematic biopsy only [10].

Biopsy specimens were evaluated locally by dedicated uropathologists according to the International Society of Urologic Pathology (ISUP) Grade Group [22,23]. CsPCa was defined as ISUP Grade Group ≥ 2 [10].

### 2.3. Predictive Variables Used in BCN-PMs and Individual csPCa Risk Estimation

BCN-PM 1 included age at biopsy (years), serum PSA level (ng/mL), DRE finding (normal vs. suspicious), family history of PCa (no vs. yes), prior negative biopsy (no vs. yes), and prostate volume estimated by DRE (small, median, or large) [15]. BCN-PM 2 included the same variables as BCN-PM 1, but replaced DRE-derived prostate volume with MRI-derived prostate volume measured (mL) and incorporated the PI-RADS v2.1 score (1–5) [15].

Individual likelihoods of csPCa were centrally calculated after data base harmonization using scripts based on the BCN-PM 1 and 2 nomograms accounting for the weighted contribution of each predictive variable (Table S1) [14,15].

### 2.4. Statistical Analysis

Anonymized datasets were harmonized across centers. Data were reported in accordance with the Standards of Reporting for MRI-targeted Biopsy Studies (START) [24]. Continuous variables are presented as medians and interquartile range (IQR), and categorical variables as frequencies. Comparisons were performed using the Mann–Whitney U test, Kruskal–Wallis test, and Pearson’s chi-square test. Odds ratios of csPCa and corresponding 95% confidence intervals (CIs) were calculated. Model calibration was assessed using calibration plots and calibration-in-the-large (CITL), calibration slope (Slope), and Brier score (Brier). When recalibration was applied, the updated intercept and slope were reported. Discrimination for csPCa was evaluated with receiver operating characteristic (ROC) curves, and areas under the curve (AUCs) were compared using DeLong’s test. Net benefit was assessed with decision curve analysis (DCA), and clinical utility curves (CUCs) were used to estimate avoided biopsies and missed csPCa across continuous threshold probabilities. Thresholds corresponding to 95% csPCa sensitivity for BCN-PM1 and 2 were used to evaluate BCM-PM 1 and 2 individually, and sequentially as BCN risk-stratified pathway (RSP), in terms of MRI avoidance, prostate biopsies, missed csPCa, and biopsy performance (csPCa detected cases per biopsies performed). A two-sided *p*-value < 0.05 was considered significant. Analyses were performed using R (version 4.3.2; R Foundation for Statistical Computing, Vienna, Austria). R scripts for constructing ROC, DCA, CUC, calibrations plots with metrics, and fixed-sensitivity thresholds are presented in Table S2.

## 3. Results

### 3.1. Characteristics of Validation Cohort

Predictive variables used in BCN-PM 1 and 2 are summarized in Table 1. Overall PCa was detected in 462 of 661 men (69.9%), being 355 csPCa (53.7%) and 107 iPCa (16.2%). ISUP Grade Group distribution was: 1 in 107 cases (16.2%), 2 in 183 cases (27.7%), 3 in 69 (10.4%), 4 in 60 (9.1%), and 5 in 43 (6.5%). PCa suspicion was primarily based on a serum PSA > 3.0 ng/mL; however, three men (0.5%) had a suspicious DRE despite a serum PSA < 3.0 ng/mL. Among additional variables, 68 men (10.3%) were receiving 5-ARI treatment. MRI was performed at 1.5 Tesla in 192 men (29.0%) and at 3 Tesla in 469 men (71.0%). MRI-TRUS fusion was software-based in 658 cases (99.5%) while a cognitive approach was used in three cases (0.5%). Biopsies were performed via the transperineal route in 656 cases (99.2%) and via the transrectal route in five (0.8%). In CAU and HCUCH between 2- and 4-core targeted biopsy and 10-core systematic biopsy were performed while mapping per 5 mm of suspicious lesions and 12-core systematic biopsy in CB.

### 3.2. Relationship Between PI-RADS Score and csPCa Detection

The relationship between the PI-RADS v2.1 category and csPCa detection is summarized in Table 2. CsPCa was detected in 4 of 54 men (7.4%) with PI-RADS 2, 35 of 145 (26.2%) with PI-RADS 3, 200 of 319 (62.7%) with PI-RADS 4, and 113 of 143 (79.0%) with PI-RADS 5.

### 3.3. Analysis of Heterogeneity Among Participant Centers

The comparative analysis of variables included in BCN-PM 1 and 2 across participating centers is presented in Table 3. With the exception of age, which was similar across centers, all other variables differed significantly, indicating heterogeneity among the study population. These findings support the need for center-specific analyses.

### 3.4. Calibration of BCN-PM 1 and 2

**Figure 2:**
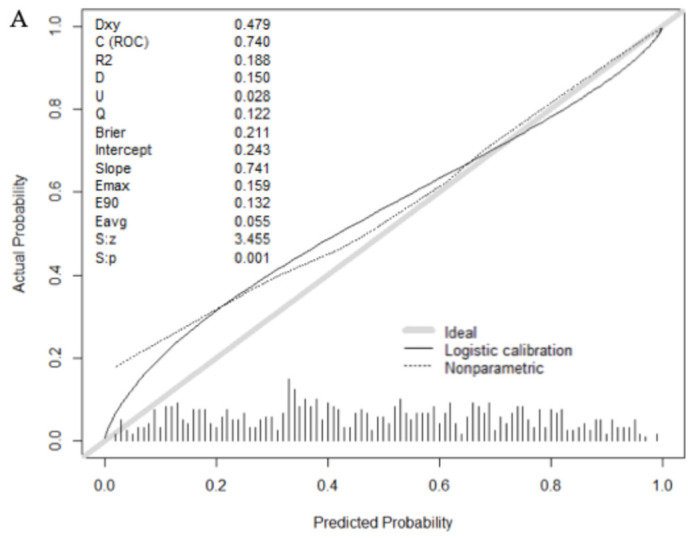

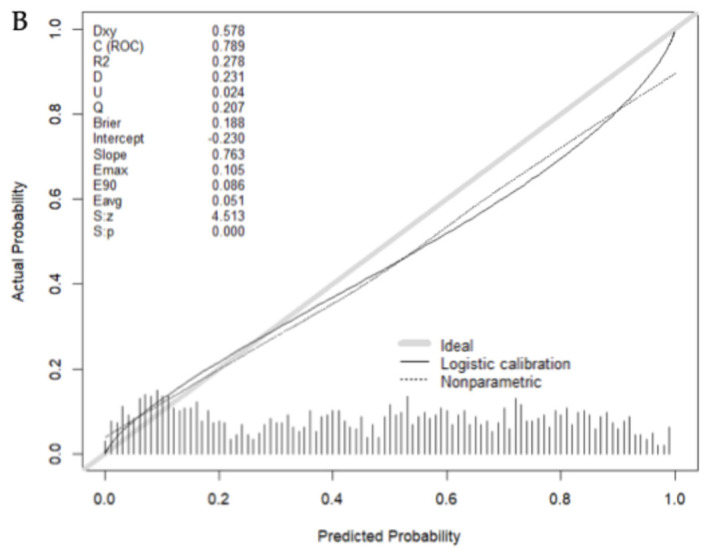
Calibration plots and metrics of BCN-PM 1 (**A**) and BCN-PM 2 (**B**).

Calibration plots are shown in Figure 2A,B. Both models demonstrated good calibration with strong agreement between predicted probabilities and observed csPCa rates. BCN-PM 1 closely followed the reference line, with minor deviations at higher predicted probabilities, whereas BCN-PM 2 showed modest departures at the extremes of risk. Calibration metrics are presented as calibration-in-the large, calibration slope, and Brier. Additionally, discrimination for csPCa is presented as the AUC of ROC curves (C/ROC).

### 3.5. Discrimination Ability of BCN-PM 1 and 2 for csPCa

**Figure 3:**
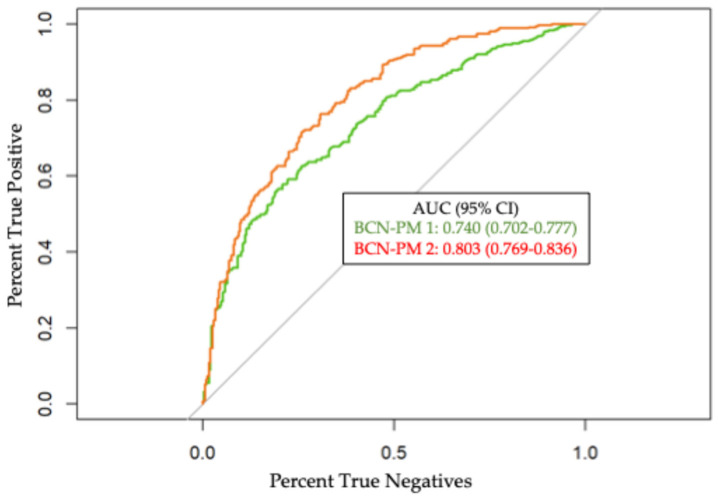
Receiver operating characteristic (ROC) curves representing the discrimination of BCN-PM 1 and BCN-PM 2 for sPCa.

The AUC of the ROC curve was 0.740 (95% CI 0.702–0.777) for BCN-PM 1 and 0.803 (0.769–0.836) for BCN-PM 2 (*p* < 0.001) (Figure 3).

### 3.6. Net Benefit of BCN-PM1 and 2 over Biopsy-All and Biopsy-None Strategies

**Figure 4:**
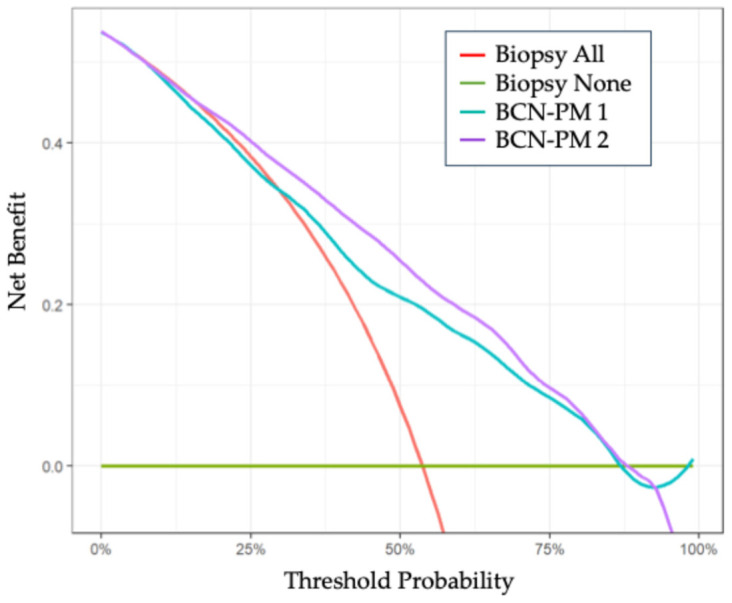
Decision curve analysis illustrating the net benefit of BCN-PM 1 and BCN-PM 2 relative to biopsy-all and biopsy-none strategies.

Both models demonstrated net benefit compared with the biopsy-all and biopsy-none strategies. BCN-PM 2 showed greater net benefit than BCN-PM 1 starting at a 19% threshold probability of csPCa (Figure 4). The net benefit of BCN-PM 2 began at a threshold probability of 19% for csPCa, whereas that of BCN-PM 2 began at 30%.

### 3.7. Clinical Utility of BCN-PM 1 and 2

**Figure 5:**
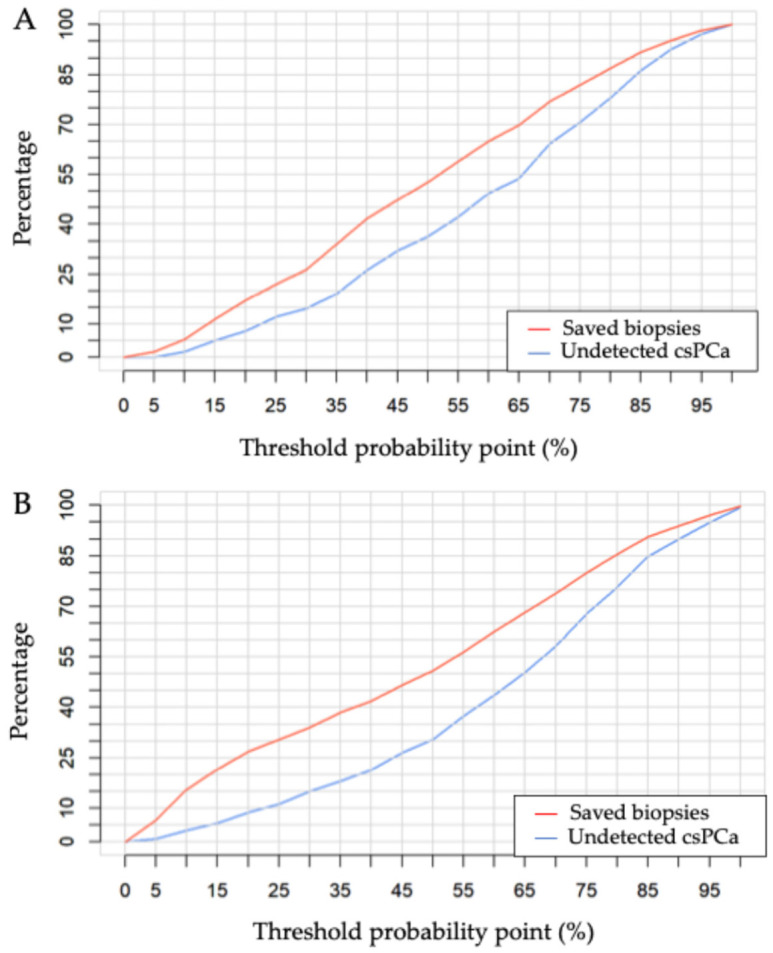
Clinical utility curves showing the proportion of avoided biopsies and undetected csPCa across a range of threshold probabilities for BCN-PM 1 (**A**) and BCN-PM 2 (**B**).

CUCs show the relationship between biopsy avoidance rates and the percentage of missed csPCa across a range of threshold probabilities for BCN-PM 1 and 2 (Figure 5A,B).

The rates of avoided biopsies and missed csPCa corresponding to each 5% increment in csPCa threshold probability (from 0% to 100) for BCN-PM 1 and BCN-PM 2 were calculated and are reported in Table S3.

As high sensitivity is required in clinical practice to ensure that only an acceptable and predictable number of csPCa cases are missed when these models are applied sequentially or individually, the clinical performance of BCN-PM 1 and BCN-PM 2 was evaluated at sensitivities of 100%, 97.5%, and 95%, as presented in Table 4. Higher specificity and biopsy avoidance rates were observed for BCN-PM 2; however, these differences were not statistically significant.

At 95% sensitivity, BCN-PM 1 avoided 70 MRI requests (10.6%), while BCN-PM 2 avoided 128 biopsies (19.4%).

### 3.8. Clinical Usefulness of the BCN Risk-Stratified Pathway

**Figure 6:**
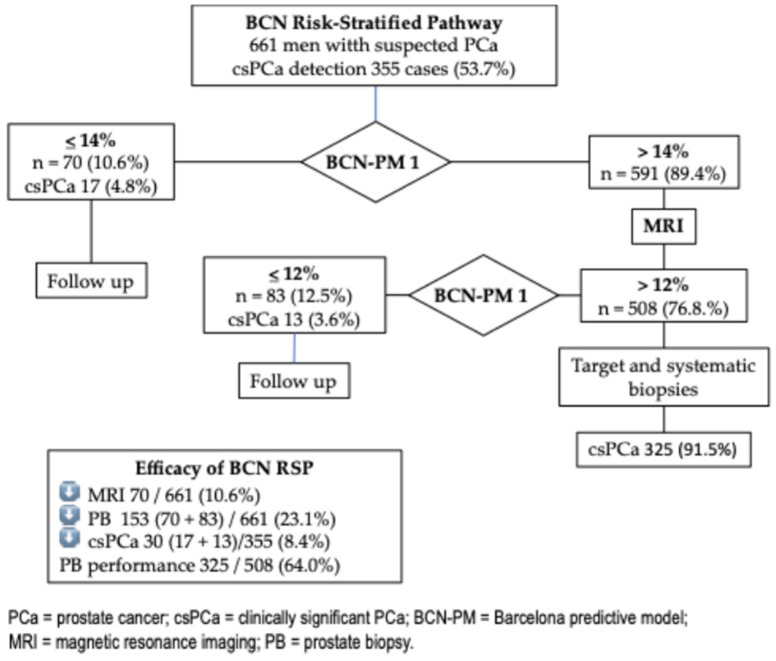
BCN risk-stratified pathway sequencing BCN-PM 1 and BCN-PM 2 at 95% sensitivity thresholds.

The sequential stratification using BCN-PM 1 and 2 at 95% sensitivity threshold is presented in Figure 6.

Initial stratification using a 14% threshold for BCN-PM 1 avoided 70 MRI requests (10.6%) at the cost of missing 17 csPCa cases (4.8%). The resulting 591 men (89.4%) with BCN-PM 1 > 14% were subsequently stratified using a 12% threshold for BCN-PM 2, avoiding 83 prostate biopsies (12.5%) while missing 13 csPCa cases (3.6%). Overall, 508 men (76.8%) underwent prostate biopsy, with 325 csPCa cases detected. The prostate biopsy yield was 64.0%, compared with 53.7% in the overall series in which all men underwent biopsy (*p* < 0.001).

### 3.9. Behavior of BCN-PM 2 According to PI-RADS Category

The performance of BCN-PM 2 according to PI-RADS score was first evaluated by analyzing model calibration within each category. Calibration plots are presented in Figure S2A–C, and calibration metrics are reported in Table S4. Calibration of BCN-PM 2 in PI-RADS 2 could not be assessed due to the small number of cases (54). Calibration was good in PI-RADS 3 and 4 whereas it was poorer in PI-RADS 5 (Figure S1). Discrimination for csPCa (ROC analysis), net benefit (DCA) and clinical utility (CUC) are shown in Figure S2A,D,G,J. The AUC in PI-RADS 2 was 0.855 (95% CI 0.729–0.981), 0.709 (95% CI 0.614–0.804) in PI-RADS 3, 0.700 (95% CI 0.643–0.758) in PI-RADS 4, and 0.649 (95% CI 0.532–0.766) in PI-RADS 5. The differences between PI-RADS 2 and 3–5 corresponded to a *p* value of 0.051, 0.020 and 0.016, respectively, whereas differences between PI-RADS 3 to 4–5 were not statistically significant (*p* > 0.05). Net benefit across PI-RADS score is presented in Figure S2B,E,H,K. CUCs are presented in Figure S2C,F,I,L. At a 95% sensitivity threshold (12%), application of BCN-PM2 avoided 44 (81.5%) undetected biopsies in PI-RADS 2, with two csPCa cases missed (50%). In PI-RADS 3, 74 biopsies (51.0%) were avoided, missing 38 csPCa cases (31.6%). In PI-RADS 4, 11 biopsies (3.4%) were avoided, with two csPCa cases missed (1.0%). In PI-RADS 5, three biopsies (2.1%) were avoided, with two csPCa cases missed (1.8%) (Table 5).

Among 355 csPCa cases detected, 18 csPCa were undetected (5.1%), and corresponded to 0.6% in PI-RADS 2, 4.2% in PI-RADS 3, 0.6% in PI-RADS 4, and 0.6% in PI-RADS 5.

### 3.10. Behavior of BCN-PM 1 and 2 According to the Participating Center

This analysis was performed due to the observed heterogeneity of predictive variables used in BCN-PM 1 and 2 across participating centers. Calibration plots are show in Figure S3A–C, and calibration metrics are summarized in Table S5. Overall, calibration of BCN-PM 1 and 2 was good in CAU and CB, and it was uncertain in HCUCH. The AUCs of ROC curves for BCN-PM 1 were 0.728 (95% CI 0.658–0.797), 0.716 (95% CI 0.716–0.822), and 0.679 (95% CI 0.593–0.765) (Figure S4A) with no statistically significant differences among centers, *p* > 0.05 (Table S6). For BCN-PM 2, the AUCs were 0.764 (95% CI 0.699–0.828) in CAU, 0.895 (95% CI 0.859–0.0932) in CB, and 0.706 (95% CI 0.623–0.788) in HCUCH (Figure S4B) with significant differences observed between CB and CAU, *p* < 0.001; between CB and HCUCH, *p* < 0.001; and no statistical differences between CAU and HCUCH, *p* > 0.05 (Table S6). Net benefit was higher in CAU and CB, and limited in HCUCH, even in BCN-PM 1 (Figure S5A–C) as in BCN-PM 2 (Figure S6A–C). According to CUC, at a 95% sensitivity threshold for csPCa, BCN-PM 1 avoided 13% of MRI requests in CAU, 14% in CB, and 10% in HCUCH (Figure S5D–F). The corresponding rates of biopsy avoidance for BCN-PM 2 were 17%, 24%, and 18%, respectively (Figure S6D–F). In summary, the behavior of BCN-PM 1 and 2 was good in CAU and CB, while in HCUCH it seemed limited.

## 4. Discussion

This study demonstrates the successful external validation of BCN-PM 1 and BCN-PM 2 in an Ibero-American population, representing a challenging setting with substantial differences from the development cohort. BCN-PM 1 and 2 were developed in a cohort of nearly 1500 men with suspected PCa who underwent prostate biopsy between 2016 and 2019 at a single academic center in Barcelona, within the framework of the opportunistic early sPCa detection program in Catalonia (Spain). They were initially externally validated in a cohort of nearly 1000 men with suspected PCa who underwent biopsy at two centers in the same metropolitan area, following the same diagnostic approach recommended at that time. Exclusion criteria included men receiving 5-ARI treatment for symptomatic benign prostatic hyperplasia due to its impact on serum PSA levels, as well as those with a previous diagnosis of PCa or atypical small acinar proliferation, given their potential influence on csPCa incidence. MRI findings were reported using PI-RADS v 2.0, and biopsies were performed via the transrectal route, obtaining two to four targeted cores per PI-RADS ≥ 3 lesion (up to three lesions), combined with a 12-core systematic biopsy using cognitive fusion imaging. All men with PI-RADS < 3 underwent a 12-core systematic biopsy [14,15].

Subsequently, BCN-PM 2 has been validated in men receiving 5-ARI treatment, who represented 10% of the current validation cohort [16], as well as in cohorts evaluated using PI-RADS v 2.1 and undergoing transperineal biopsy, which is now the standard approach for csPCa diagnosis [18]. In addition, BCN-PM 2 has been validated within a federated network across three European countries [18]. Across these studies, MRI scanners opera-ting at 1.5 and 3 Tesla were used, along with software-based MRI-TRUS fusion imaging techniques, including newer biopsy protocols targeting both lesional and perilesional areas [25].

The cohort of 661 men with suspected PCa included in this head-to-head validation of BCN-PM 1 and 2 was selected from 2017 men undergoing opportunistic early detection of PCa in 2025, all of whom had complete data for the variables included in both predictive models. DRE-derived prostate volume was available only at the three centers participating in this external validation. All men with suspected PCa, identified primarily on the basis of a serum PSA > 3.0 ng/mL and/or suspicious DRE, were managed according to current criteria aimed at optimizing the opportunistic early detection of csPCa [10]. Two major differences were observed between the development and validation cohorts. First, fewer than 5% of men undergoing biopsy had PI-RADS < 3 lesions, compared with nearly 25% in the development cohort. This difference is explained by the fact that between 2016 and 2019, all men with PI-RADS < 3 underwent systematic biopsies. Second, the csPCa detection rate was 53.7% in this validation cohort, compared with less than 40% in the development cohort [14,15]. This likely reflects a higher-risk profile of the validation cohort and a more challenging setting for model validation. Nonetheless, the csPCa detection rates within each PI-RADS version 2.1 category fell within the 95% confidence intervals reported in a meta-analysis, supporting the representativeness of the present validation cohort [21].

Both models demonstrated appropriate calibration across the risk spectrum. BCN-PM 1 slightly overestimated risk at higher predicted probabilities, whereas BCN-PM 2 showed modest departures at the extremes. Both models also showed adequate discrimination for csPCa, with an AUC and corresponding 95% confidence intervals consistently above 0.700 [26]. In decision curve analysis, both models provided net benefit compared with biopsy-all and biopsy-none strategies, although the benefit was greater for BCN-PM 2 [27]. Clinically, the most relevant finding was that, at a sensitivity of 95% for csPCa detection, BCN-PM 1 avoided 70 MRI requests (10.6%), while BCN-PM 2 avoided 128 prostate biopsies (19.4%) [28].

Although the proportion of men with PI-RADS ≤ 3 was very low compared with the development cohort, likely due to preselection of higher-risk men for biopsy based on an elevated PSA density, suspicious DRE, or rising PSA after a negative biopsy, the performance of BCN-RSP was also evaluated. MRI requests decreased by 10.6% and prostate biopsies were reduced by 23.1% at the cost of missing 8.4% of csPCa cases. The diagnostic yield of prostate biopsy improved from 53.7% to 64% after the application of sequenced BCN-PM 1 and 2. These results suggest that sequential application of predictive models enhances performance compared with their independent application [13].

Analysis of BCN-PM 2 performance according to PI-RADS category provides insight into its clinical application. As we observed in both the development cohort and initial external validation, achieving 100% sensitivity in PI-RADS 5 lesions was not feasible; therefore, missing any csPCa case in this category is clinically unacceptable. At 95% sensitivity, 1.8% of csPCa cases were missed while only 2.1% of biopsies were avoided. In PI-RADS 4, performance was limited, with 1% of csPCa cases missed and 3.4% of biopsies avoided. In PI-RADS 3, 31.8% of csPCa cases were missed while 51% of biopsies were avoided. Finally, in the preselected PI-RADS 2, 50% of csPCa cases were detected while 81.5% of biopsies were avoided. Overall, 18 csPCa cases were missed (5.1%), distributed as 0.6% in PI-RADS 2, 4.2% in PI-RADS 3, 0.6% in PI-RADS 4, and 0.6% in PI-RADS 5 [14].

Given that heterogeneity was observed in the predictive variables used by BCN-PM 1 and 2, as well as in csPCa detection rates across participating centers, individual calibration and analyses of clinical utility metrics were performed at each center. Calibration was satisfactory in CAU and CB but less optimal in HCUCH. Discrimination for csPCa with BCN-PM 1 was statistically similar across centers but it was highest in CB, where the detection rate was 61.8% compared with 47.8% in CAU and 45% at HCUCH. This difference may be attributable to a more aggressive biopsy protocol at CB [25]. Across all centers, both models demonstrated net benefit, with reductions of 10–14% in MRI requests and 17–24% in prostate biopsies. Overall, heterogeneity translated into varying degrees of clinical utility across centers, and satisfactory validation.

Strengths of this validation include prior evaluations of BCN-PM 2 in men receiving 5-ARI therapy, allowing inclusion of these patients in the current study, as well as validation in cohorts using PI-RADS v2.1 and transperineal biopsy, which reflect current clinical practice. The clinical utility of BCN-PM 2 was also assessed by PI-RADS category, demonstrating that its use in men with PI-RADS 5 is not advisable. In contrast, a key limitation is the lack of inclusion of prior external validation for BCN-PM 1. Additional limitations include the relatively small sample size for this head-to-head comparison, which may introduce selection bias, as well as the reliance on DRE-derived prostate volume, which is not routinely measured prior to MRI [29]. The low proportion of PI-RADS < 3 cases reflects current recommendations to biopsy only high-risk men, based on PSA density, suspicious DRE finding, PSA kinetics prior to negative biopsy, or patient preference [10]. The absence of centralized MRI interpretation and pathological review may also be considered a limitation, although it reflects real-world clinical practice. Finally, intercenter heterogeneity and minor variability in diagnostic protocols may have influenced results.

Predictive models require freely and accessible risk calculators to facilitate clinical implementation [13]. External validation is essential in populations where these models are intended to be applied or diagnostic practices evolve [26]. Artificial intelligence increasingly assists in PCa diagnosis [30]. Dynamic updating of predictive models remains an ongoing challenge [31]. In the near future, the application of machine learning algorithms within federated networks may enable continuous validation and updating of predictive models, ensuring robust and generalizable performance across multiple sites [32]. Further validation in larger and more diverse Ibero-American populations is warranted to confirm these findings.

## 5. Conclusions

BCN-PM 1 and BCN-PM 2 were successfully validated in an Ibero-American population. BCN-PM 1 was able to reduce the number of MRI requests, whereas BCN-PM 2 decreased the rate of unnecessary prostate biopsies, with an acceptable trade-off in missed csPCa. Both predictive models appear suitable for implementation.

## Figures and Tables

**Table 1:** Characteristics of variables used by BCN-PM 1 and 2.

Variable	Measurement
Number of men, n	661
Median age, years (IQR)	66 (61–72)
Median serum PSA, ng/mL (IQR)	6.8 (5.0–9.7)
Abnormal DRE, n (%)	98 (14.8)
Prior negative biopsy, n (%)	120 (18.2)
Family history of PCa, n (%)	66 (10.0)
Prostate volume derived from DRE, n (%)	
Small, n (%)	271 (41.0)
Median, n (%)	251 (38.0)
Large, n (%)	139 (21.0)
Median prostate volume derived from MRI, mL (IQR)	50 (35–65)
PI-RADS score	
1, n (%)	0 (0.0)
2, n (%)	54 (8.2)
3, n (%)	145 (21.9)
4, n (%)	319 (48.3)
5, n (%)	143 (21.6)
Overall detection of csPCa, n (%)	355 (53.7)

n = number; IQR = interquartile range; PSA = prostate-specific antigen; DRE = digital rectal examination; MRI = magnetic resonance imaging; PI-RADS = Prostate Imaging–Reporting and Data System; csPCa = clinically significant PCa.

**Table 2:** Relationship between PI-RADS v2.1 category and csPCa detected rate in prostate biopsy in the study cohort for validation of BCN-PM 1 and 2.

Detection of csPCa	PI-RADS Score					All, n (%)
	1	2	3	4	5	
csPCa, n (%)	0 (0.0)	4 (7.4)	38 (26.2)	200 (62.7)	113 (79.0)	355 (53.7)
All cases, n (%)	0 (0.0)	54 (4.8)	145 (21.9)	319 (48.3)	143 (21.0)	661 (100)

PI-RADS = Prostate Imaging–Reporting and Data System; csPCa = clinically significant PCa.

**Table 3:** Analysis of heterogeneity among participant centers.

Variable	CAU	CB	HCUCH	*p* Value
Number of men, n	201	309	151	-
Median age, years (IQR)	66 (55–71)	67 (56–72)	67 (54–72)	0.703
Median serum PSA, ng/mL (IQR)	7.0 (5.6–10.6)	6.3 (4.2–9.4)	6.6 (4.0–9.5)	0.043
Abnormal DRE, n (%)	22 (10.9)	47 (15.2)	29 (19.2)	0.054
Prior negative biopsy, n (%)	38 (18.9)	75 (24.3)	7 (4.6)	<0.001
Family history of PCa, n (%)	20(10.0)	32 (10.4)	14 (9.3)	0.389
Prostate volume derived from DRE, n (%)				
Small, n (%)	64 (31.8)	159 (51.5)	48 (31.8)	
Median, n (%)	91 (45.3)	96 (31.0)	64 (42.4)	<0.001
Large, n (%)	46 (22.9)	54 (17.5)	39 (25.8)	
Median prostate volume derived from MRI, mL (IQR)	50 (30–68)	47 (25–68)	49 (25–63)	0.037
PI-RADS score				
2, n (%)	0 (0)	48 (15.5)	6 (4.0)	
3, n (%)	52 (25.9)	71 (23.0)	22 (14.6)	
4, n (%)	116 (57.7)	118 (38.2)	85 (56.3)	<0.001
5, n (%)	33 (16.4)	72 (23.3)	38 (25.1)	
Overall detection of csPCa, n (%)	96 (47.8)	191 (61.8)	68 (45.0)	<0.001

CAU = Centro Argentino de Urología; CB = Clinica Creu Blanca; HCUCH = Hospital Clínico de la Universidad de Chile; n = number; IQR = interquartile range; PSA = prostate-specific antigen; DRE = digital rectal examination; MRI = magnetic resonance imaging; PI-RADS = Prostate Imaging–Reporting and Data System; csPCa = clinically significant PCa.

**Table 4:** Thresholds, corresponding specificities and biopsy avoidance rates at sensitivities of 100%, 97.5%, and 95% for BCN-PM 1 and BCN-PM 2.

Parameter	BCN-PM 1	BC-PM 2	*p*-Value
**Sensitivity target, %**	**100**	**100**	
Threshold, %	5.4	3.5	
Sensitivity observed, %	100	100	
Specificity (95% CI), %	3.6 (1.6–5.9)	8.5 (5.6–11.8)	=0.954
Positive predictive value, %	54.6	55.9	
Negative predictive value, %	100	100	
True positives, n	355	355	
True negatives, n	11	26	
False positives, n	295	280	
False negatives, n	0	0	
Saved MRI/biopsies, n	11	26	
Undetected csPCa	0	0	
**Sensitivity target, %**	**97.5**	**97.5**	
Threshold, %	11.1	8.8	
Sensitivity observed, %	97.7	97.7	
Specificity (95% CI), %	10.8 (7.7–14.4)	25.2 (20.5–30.3)	=0.972
Positive predictive value, %	56.0	60.2	
Negative predictive value, %	80.5	90.6	
True positives, n	347	347	
True negatives, n	33	77	
False positives, n	273	229	
False negatives, n	8	8	
Saved MRI/biopsies, n	41	45	
Undetected csPCa	8	8	
**Sensitivity target, %**	**95**	**95**	
Threshold, %	14.0	12.0	
Sensitivity observed, %	95.2	95.2	
Specificity (95% CI), %	17.3 (13.4–21.5)	36.3 (31.3–41.4)	=0.993
Positive predictive value, %	57.2	63.4	
Negative predictive value, %	75.7	86.7	
True positives, n	338	338	
True negatives, n	53	111	
False positives, n	253	195	
False negatives, n	17	17	
Saved MRI/biopsies, n	70	128	
Undetected csPCa	17	17	

CI = confidence interval; MRI = magnetic resonance imaging; csPCa = clinically significant prostate cancer.

**Table 5:** Avoided prostate biopsies and missed csPCa after applying BCN-PM2 at a 95% sensitivity threshold according to PI-RADS categories.

PI-RADS Score	Avoided Biopsies n (%)	Missed csPCa n (%)	Odds Ratio (95% CI)	*p* Value
2	44/54 (81.5)	2/4 (50)	5.2 (0.6–42.9)	0.092
3	74/145 (51.0)	12/38 (31.8)	2.9 (1.3–6.5)	0.005
4	11/319 (3.4)	2/200 (1.0)	8.1 (1.7–38.2)	0.002
5	3/143 (2.1)	2/113 (1.8)	1.9 (0.2–21.8)	0.595

PI-RADS = Prostate Imaging–Reporting and Data System; csPCa = clinically significant prostate cancer; CI = confidence interval.

## Data Availability

The data presented in this study are available on request from the corresponding author.

## Supplementary Materials

The following supporting information can be downloaded at: https://www.mdpi.com/article/10.3390/cancers18111810/s1, Figure S1: Calibration plots of BCN-PM 2 according to the PI-RADS category 3 (A), 4 (B), and 5 (C); Figure S2: Discrimination of csPCa (ROC and AUC [95% CI]), net benefit (DCA) and clinical utility (CUC) of BCN-PM 1 according to the PI-RADS category: 2 (A–C), 3 (D–F), 4 (G–J), and 5 (J–L); Figure S3: Calibration plots of BCN-PM 1 and BCN-PM 2 in participating centers. BCN-PM 1 in CAU (A), CB (B) and HCUCH (C), and BCN-PM 2 in CAU (D), CB (E), and HBUCH (F); Figure S4: Discrimination of csPCa (ROC and AUC [95% CI]) at each participant center of BCN-PM 1 (A), and BCN-PM 2 (B); Figure S5: Calibration metrics of BCN-PM 1 and 2 by participant centers; Figure S6: Bootstrap-based AUC DeLong pairwise comparison by center; Table S1: Scripts in R to calculate the probability of csPCa by the BCN-PM 1 and 2 from nomograms coefficients; Table S2: Scripts in R to construct ROC, DCA, CUC, calibration, and fixed-sensitivity thresholds; Table S3: Percentage of undetected csPCa and saved biopsies according to a 5% increased threshold for the BCN-PM 1 and BCN-PM 2; Table S4: Calibration metrics of BCN-PM 2 according to the PI-RADS categories; Table S5: Calibration metrics of BCN-PM 1 and 2 by participant centers; Table S6: Bootstrap-based AUC DeLong pairwise comparison by center.

## Abbreviations

The following abbreviations are used in this manuscript:

AUC	Area under the curve
BCN-PM	Barcelona predictive model
CI	Confidence interval
CUC	Clinical utility curve
DCA	Decision curve analysis
DRE	Digital rectal examination
iPCa	Insignificant PCa
IQR	Interquartile range
MRI	Magnetic resonance imaging
PCa	Prostate cancer
PI-RAS	Prostate Imaging–Reporting and Data System
PSA	Prostate-specific antigen
ROC	Receiver operating characteristic
sPCa	Significant PCa
TRUS	Transrectal ultrasound
