# Supplementary material for: External Validation and Clinical Impact of the Barcelona Predictive Models for Detecting Significant Prostate Cancer in Prostate Biopsies in an Ibero-American Population
Source: Cancers (Basel). 2026 Jun 1;18(11):1810. doi: 10.3390/cancers18111810 (PMC13255904; doi:10.3390/cancers18111810)
Supplement: Supplementary file 1 [file cancers-18-01810-s001.zip › cancers-4301170-supplementary.pdf]

Table S1. Scripts in R to calculate the probability of csPCa by the BCN-PM 1 and 2 from nomograms coefficients.

```
# BCN1 variables: Age (ED), PSA, DRE (TR), TB, AF (FH), PVRE (VPTR)
# BCN2 variables: Age (ED), PSA, DRE (TR), TB, AF (FH), PV, PIRADS (PIR)
# Output: probability of significant prostate cancer (sPCa), range [0, 100]%
# ============================================================
f_RC_BCN1 <- function(x) {
  1 / (1 + exp(-(-6.59480804 +
    0.07748286   * Data$Age[x] +
    2.56201433   * log(Data$PSA[x], 10) -
    1.05181425   * (if (Data$PVRE[x] == 2) 1 else 0) -
    2.62040923   * (if (Data$PVRE[x] == 3) 1 else 0) +
    1.15925385   * (if (Data$DRE[x]   == 1) 1 else 0) +
    0.52443644   * (if (Data$AF[x]    == 1) 1 else 0) -
    0.46522816   * (if (Data$TB[x]    == 2) 1 else 0)
  ))) * 100
}

f_RC_BCN2 <- function(x) {
  1 / (1 + exp(-(-6.09722628 +
    0.05465533   * Data$Age[x] +
    0.08145515   * Data$PSA[x] -
    0.03002868   * Data$PV[x] +
    0.54803581   * (if (Data$DRE[x]      == 1)   1 else 0) +
    0.58142810   * (if (Data$AF[x]       == 1)   1 else 0) -
    0.40263407   * (if (Data$TB[x]       == 2)   1 else 0) +
    1.19728849   * (if (Data$PIRADS[x] == "2") 1 else 0) +
    1.87960323   * (if (Data$PIRADS[x] == "3") 1 else 0) +
    3.46847431   * (if (Data$PIRADS[x] == "4") 1 else 0) +
    4.32641717   * (if (Data$PIRADS[x] == "5") 1 else 0)
  ))) * 100
}

# Usage:
#    Data <- your_dataset     # must contain the variables above
#    Prob_BCN1 <- sapply(seq_len(nrow(Data)), f_RC_BCN1)
#    Prob_BCN2 <- sapply(seq_len(nrow(Data)), f_RC_BCN2)
```

Table S2. Scripts in R to construct ROC, DCA, CUC, calibration, and fixed-sensitivity thresholds.

```
# Dependencies: pROC, dcurves, rms, tidyverse
# ============================================================================

# ,îÄ,îÄ Confusion matrix ,îÄ,îÄ,îÄ,îÄ,îÄ,îÄ,îÄ,îÄ,îÄ,îÄ,îÄ,îÄ,îÄ,îÄ,îÄ,îÄ,îÄ,îÄ,îÄ,îÄ,îÄ,îÄ,îÄ,îÄ,îÄ,îÄ,îÄ,îÄ,îÄ,îÄ,îÄ,îÄ,îÄ,îÄ,îÄ,îÄ,îÄ,îÄ,îÄ,îÄ,îÄ,îÄ,îÄ,îÄ,îÄ,îÄ,îÄ,îÄ,îÄ,îÄ,îÄ,îÄ,îÄ,îÄ,îÄ,îÄ,îÄ,îÄ
get_confusion <- function(prob, status, threshold) {
  pred <- ifelse(prob >= threshold, 1, 0)
  TP <- sum(pred == 1 & status == 1, na.rm = TRUE)
  TN <- sum(pred == 0 & status == 0, na.rm = TRUE)
  FP <- sum(pred == 1 & status == 0, na.rm = TRUE)
  FN <- sum(pred == 0 & status == 1, na.rm = TRUE)
  list(TP=TP, TN=TN, FP=FP, FN=FN,
       Se=TP/(TP+FN), Sp=TN/(TN+FP),
       PPV=ifelse((TP+FP)==0,NA,TP/(TP+FP)),
       NPV=ifelse((TN+FN)==0,NA,TN/(TN+FN)))
}

# ,îÄ,îÄ Highest threshold meeting Se >= target ,îÄ,îÄ,îÄ,îÄ,îÄ,îÄ,îÄ,îÄ,îÄ,îÄ,îÄ,îÄ,îÄ,îÄ,îÄ,îÄ,îÄ,îÄ,îÄ,îÄ,îÄ,îÄ,îÄ,îÄ,îÄ,îÄ,îÄ,îÄ,îÄ,îÄ,îÄ,îÄ,îÄ,îÄ,îÄ,îÄ
find_threshold_for_sensitivity <- function(prob, status, se_target,
                                           resolution = 0.001) {
  for (th in rev(seq(0, 1, by = resolution))) {
    cm <- get_confusion(prob, status, th)
    if (!is.nan(cm$Se) && cm$Se >= se_target) return(th)
  }
  return(0)
}

# ,îÄ,îÄ Bootstrap CI for specificity ,îÄ,îÄ,îÄ,îÄ,îÄ,îÄ,îÄ,îÄ,îÄ,îÄ,îÄ,îÄ,îÄ,îÄ,îÄ,îÄ,îÄ,îÄ,îÄ,îÄ,îÄ,îÄ,îÄ,îÄ,îÄ,îÄ,îÄ,îÄ,îÄ,îÄ,îÄ,îÄ,îÄ,îÄ,îÄ,îÄ,îÄ,îÄ,îÄ,îÄ,îÄ,îÄ,îÄ,îÄ,îÄ,îÄ
bootstrap_metrics <- function(prob, status, threshold, n_boot=1000, seed=42) {
  set.seed(seed); n <- length(status); sp_vals <- numeric(n_boot)
  for (i in seq_len(n_boot)) {
    idx <- sample(n, replace=TRUE)
    sp_vals[i] <- get_confusion(prob[idx], status[idx], threshold)$Sp
  }
  list(Sp_lower=quantile(sp_vals,0.025,na.rm=TRUE),
       Sp_upper=quantile(sp_vals,0.975,na.rm=TRUE))
}
```

```
# ‚îÄ‚îÄ CUC plot and table
‚îÄ‚îÄ‚îÄ‚îÄ‚îÄ‚îÄ‚îÄ‚îÄ‚îÄ‚îÄ‚îÄ‚îÄ‚îÄ‚îÄ‚îÄ‚îÄ‚îÄ‚îÄ‚îÄ‚îÄ‚îÄ‚îÄ‚îÄ‚îÄ‚îÄ‚îÄ‚îÄ‚îÄ‚îÄ‚îÄ‚îÄ‚îÄ‚îÄ‚îÄ‚îÄ‚îÄ
‚îÄ‚îÄ‚îÄ‚îÄ‚îÄ‚îÄ‚îÄ‚îÄ‚îÄ‚îÄ‚îÄ‚îÄ‚îÄ‚îÄ‚îÄ‚îÄ‚îÄ‚îÄ‚îÄ‚îÄ
# Returns data.frame with Threshold_pct, Undetected_Reclassification_pct/n,
# Saved_Biopsy_pct/n
## Based on the https://cran.r-project.org/web/packages/CUtools/index.html

CUC <- function(Prob, Status, step=5, plot_title="",
                col_ub="cornflowerblue", col_sb="tomato") {
  Prob <- as.numeric(Prob); Status <- as.numeric(Status)
  n_total <- length(Status); n_pos <- sum(Status, na.rm=TRUE)
  Th <- seq(0, 100, by=step)
  UB <- sapply(Th, function(x) sum(Status[Prob*100 < x],na.rm=TRUE)/n_pos*100)
  SB <- sapply(Th, function(x) sum(Prob*100 < x,na.rm=TRUE)/n_total*100)
  plot(Th, UB, type="l", lwd=1.5, col=col_ub,
       xlab="Threshold probability (%)", ylab="Percentage",
       ylim=c(0,100), xlim=c(0,100), main=plot_title,
       tck=1, fg="lightgray", xaxp=c(0,100,20), yaxp=c(0,100,20))
  lines(Th, SB, lwd=1.5, col=col_sb)
  data.frame(Threshold_pct=Th,
             Undetected_Reclassification_pct=round(UB,2),
             Undetected_Reclassification_n=round(UB/100*n_pos),
             Saved_Biopsy_pct=round(SB,2),
             Saved_Biopsy_n=round(SB/100*n_total))
}

# ‚îÄ‚îÄ Calibration metrics from val.prob()
‚îÄ‚îÄ‚îÄ‚îÄ‚îÄ‚îÄ‚îÄ‚îÄ‚îÄ‚îÄ‚îÄ‚îÄ‚îÄ‚îÄ‚îÄ‚îÄ‚îÄ‚îÄ‚îÄ‚îÄ‚îÄ‚îÄ‚îÄ‚îÄ‚îÄ‚îÄ‚îÄ‚îÄ‚îÄ‚îÄ‚îÄ‚îÄ‚îÄ‚îÄ‚îÄ‚îÄ
‚îÄ
# Returns CITL (intercept), slope, Brier score, C-statistic
calibration_metrics <- function(prob, status, label="") {
  vp <- rms::val.prob(prob, status, pl=FALSE)
  tibble::tibble(Group=label,
                 CITL   = round(as.numeric(vp["Intercept"]),3),
                 Slope  = round(as.numeric(vp["Slope"]),3),
                 Brier  = round(as.numeric(vp["Brier"]),3),
                 C_stat = round(as.numeric(vp["C (ROC)"]),3))
}
```

Table S3. Percentage of undetected csPCa and saved biopsies according to a 5% increased threshold for the BCN-PM 1 and BCN-PM 2.

Threshold (%)	BCN-PM 1		BCN-PM 2	
	Undetected csPCa (%)	Saved biopsies (%)	Undetected csPCa (%)	Saved biopsies (%)
0	0	0	0	0
5	0	1.66	0,85	6,51
10	1.69	5.30	3,38	15,43
15	5.07	11.50	5,63	21,63
20	7.89	17.10	8,73	26,93
25	12.11	21.94	11,27	30,56
30	14.64	26.32	14,93	34,04
35	19.15	34.04	18,03	38,43
40	26.19	41.60	21,41	41,91
45	32.11	47.35	26,48	46,6
50	36.33	52.65	30,42	50,98
55	42.25	59.00	37,46	56,43
60	49.29	65.05	43,66	62,48
65	53.80	69.74	50,42	68,23
70	64.23	76.85	58,31	73,83
75	70.70	81.85	67,89	80,03
80	78.03	86.84	75,77	85,48
85	86.20	91.68	84,79	90,62
90	92.68	95.31	89,86	93,8
95	97.18	98.34	94,93	96,97
100	100	100	99,44	99,7

csPCa = clinically significant PCa.

Table S4. Calibration metrics of BCN-PM 2 according to the PI-RADS categories.

Group	N	N sPCa+	CITL	Slope	Brier	C stat Note
BCN2 PI-RADS 2	54	4	NA	NA	NA	NA Too few cases
BCN2 PI-RADS 3	145	38	0.786	1.034	0.185	0.709
BCN2 PI-RADS 4	319	200	0.459	0.803	0.217	0.699
BCN2 PI-RADS 5	143	113	1.138	0.129	0.175	0.646

Interpretation guide: CITL (calibration-in-the-lange): 0=perfect; positive = model underestimates risk on average; negative = overestimates.Slope: 1 = perfect; <1 = predictions too extreme; >1 = predictions too conservative. Brier score: lower is better; null model (predict prevalence for all) gives reference.

Table S5. Calibration metrics of BCN-PM 1 and 2 by participant centers.

Model	Centre	N	N sPCa+	CITL	Slope	Brier	C_stat Note
BCN1	CAU	201	96	0.088	0.787	0.210	0.728
BCN1	CB	309	191	0.548	0.887	0.200	0.769
BCN1	HCUCH	151	68	-0.144	0.456	0.236	0.679
BCN2	AU	201	96	0.065	0.752	0.199	0.764
BCN2	CB	309	191	1.303	1.205	0.160	0.895
BCN2	HCUCH	151	68	-0.243	0.384	0.232	0.697

Interpretation guide: CITL (calibration-in-the-lange): 0 = perfect; positive = model underestimates risk on average; negative = overestimates. Slope: 1 = perfect; <1 = predictions too extreme; >1 = predictions too conservative. Brier score:lower isbetter; null model (predict prevalence for all) gives reference.

Table S6. Bootstrap-based AUC DeLong pairwise comparison by center.

Model	Centre_A	N_A	AUC_A	Centre_B	N_B	AUC_B	Z	p_value
BCN1	CAU	201	0.728	CB	309	0.769	-0.895	0.3710
BCN1	CAU	201	0.728	HCUCH	151	0.679	0.817	0.4137
BCN1	CB	309	0.769	HCUCH	151	0.679	1.806	0.0709
BCN2	CAU	201	0.764	CB	309	0.895	-3.594	0.0003
BCN2	CAU	201	0.764	HCUCH	151	0.706	1.049	0.294
BCN2	CB	309	0.895	HCUCH	151	0.706	0.167	0.0000

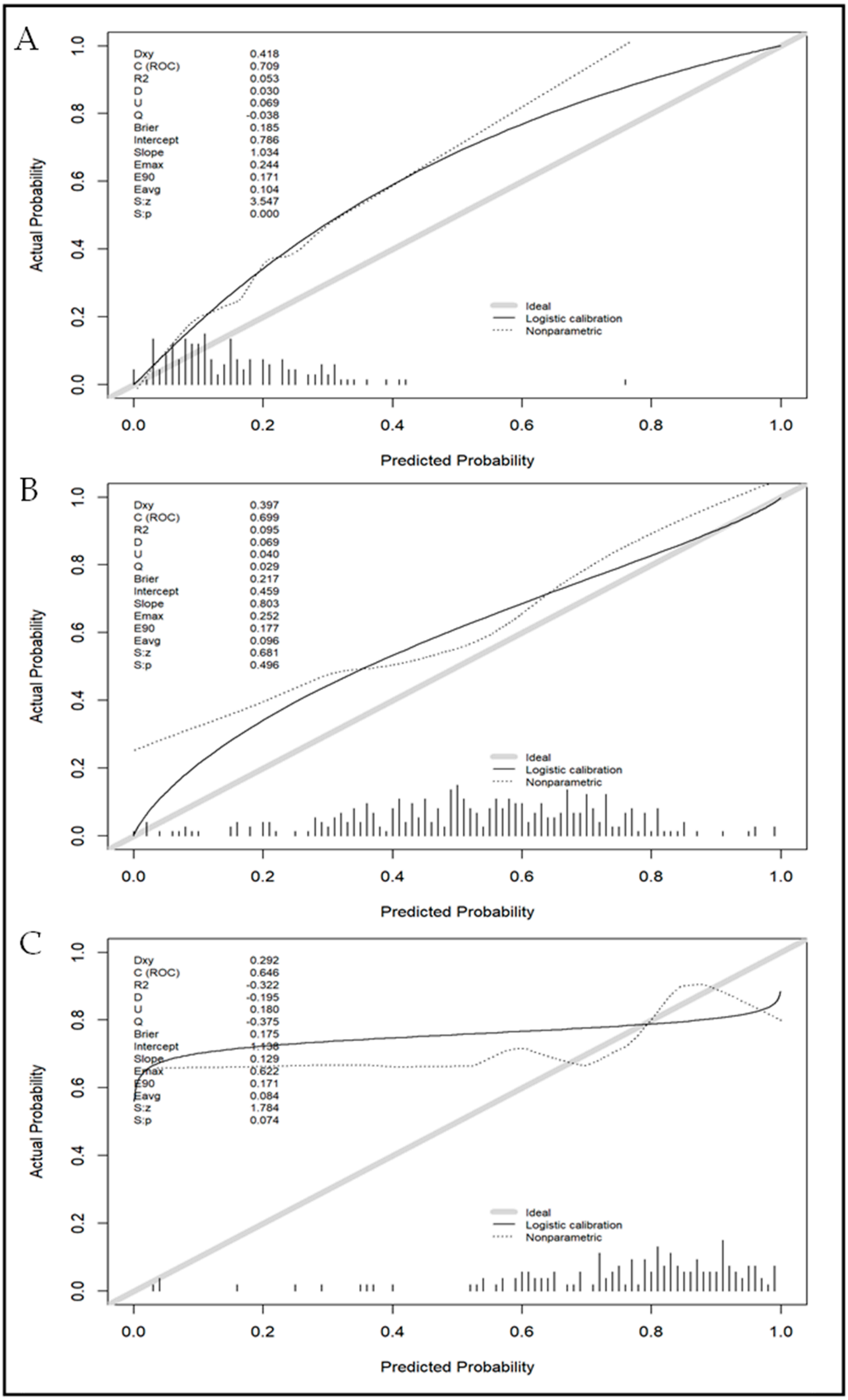

Figure S1. Calibration plots of BCN-PM 2 according to the PI-RADS category 3 (A), 4 (B), and 5 (C).

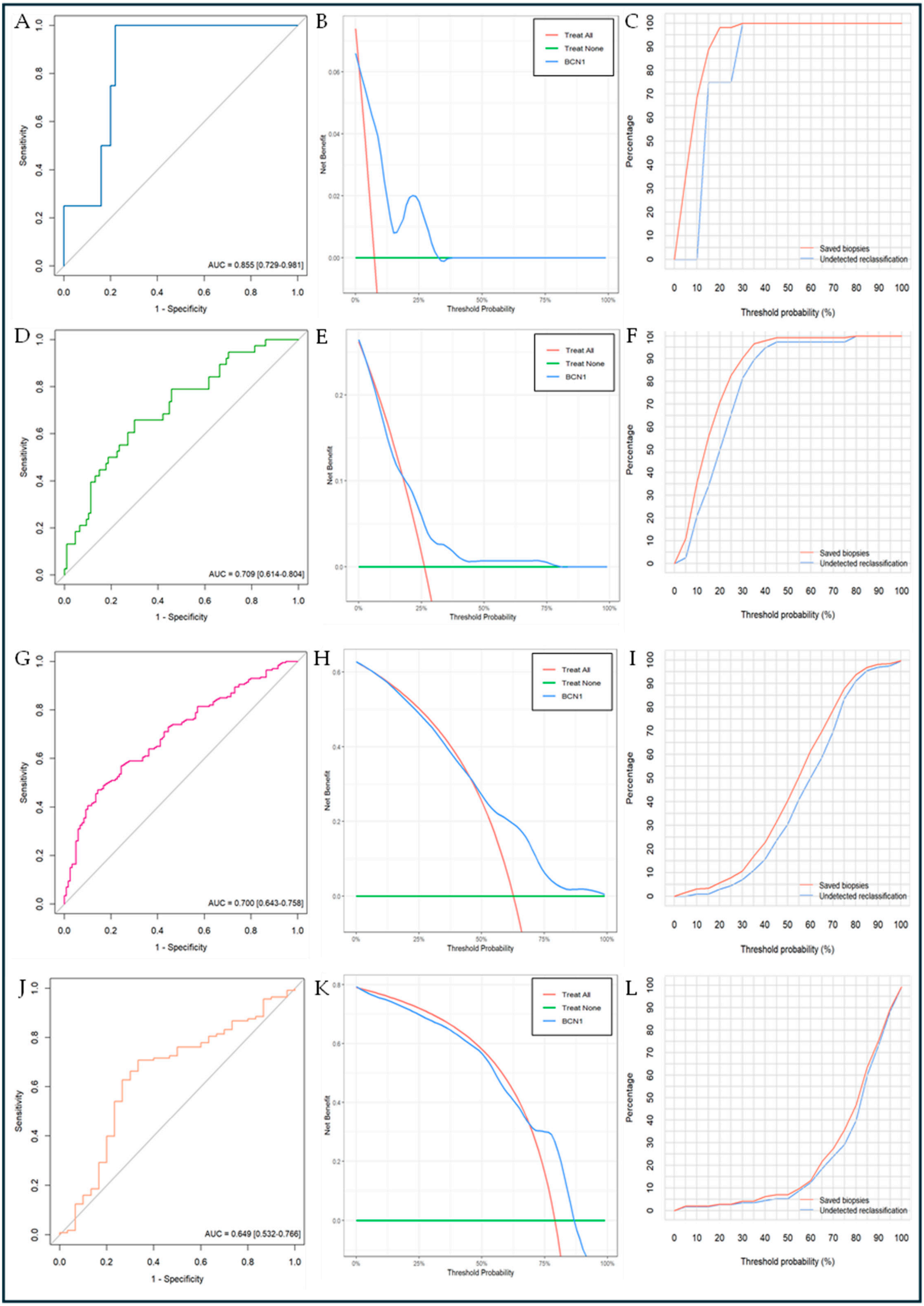
A
B
C
D
E
F
G
H
I
J
K
L
Sensitivity
1 - Specificity
AUC = 0.855 [0.729-0.981]
AUC = 0.709 [0.614-0.804]
AUC = 0.700 [0.643-0.758]
AUC = 0.649 [0.532-0.766]
Net Benefit
Threshold Probability
Treat All
Treat None
BCN1
Percentage
Threshold probability (%)
Saved biopsies
Undetected reclassification

Figure S2. Discrimination of csPCa (ROC and AUC [95% CI]), net benefit (DCA) and clinical utility (CUC) of BCN-PM 1 according to the PI-RADS category: 2 (A-C), 3 (D-F), 4 (G-J), and 5 (J-L).

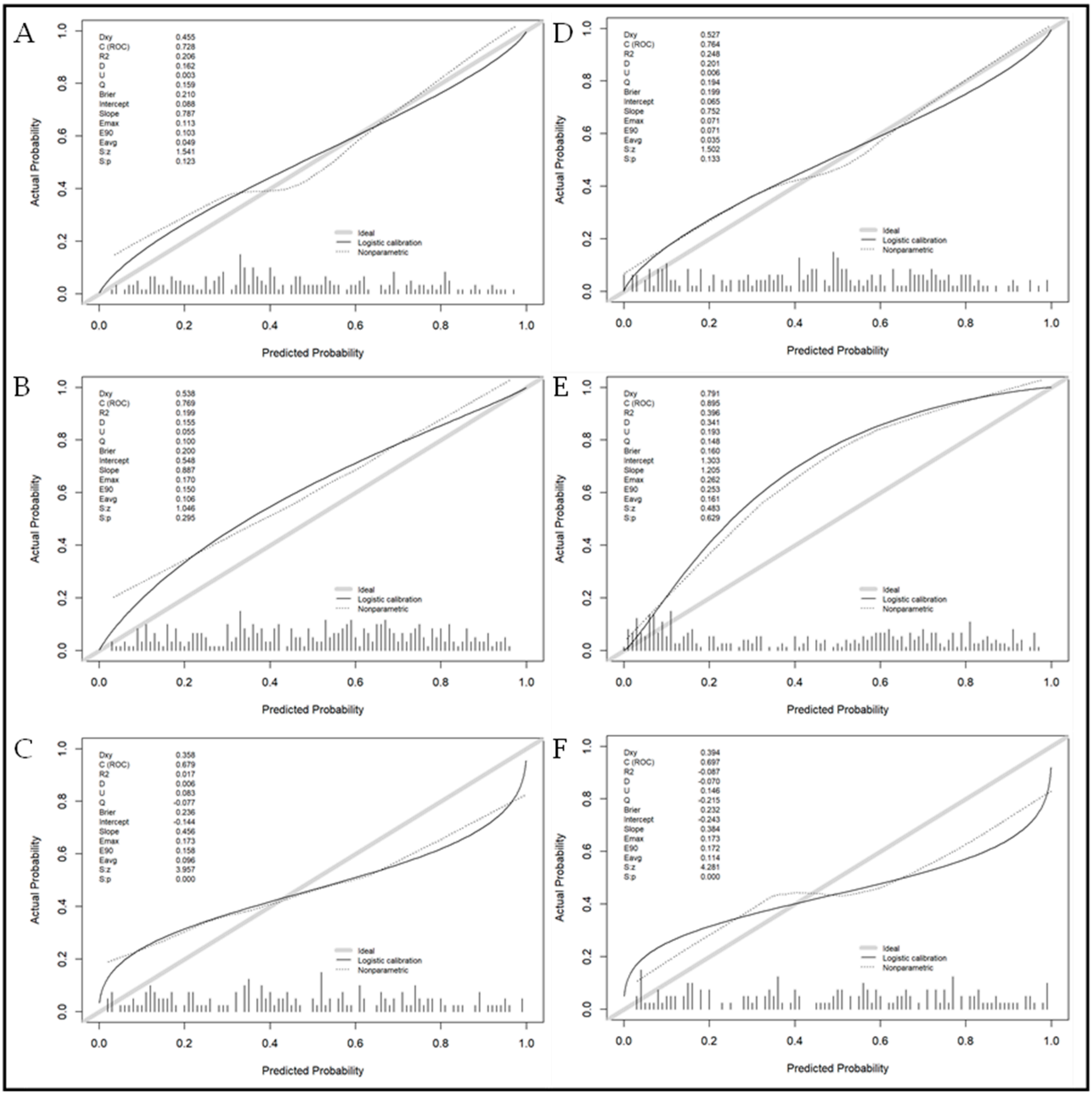

Figure S3. Calibration plots of BCN-PM 1 and BCN-PM 2 in participating centers. BCN-PM 1 in CAU (A), CB (B) and HCUCH (C), and BCN-PM 2 in CAU (D), CB (E), and HBUCH (F).

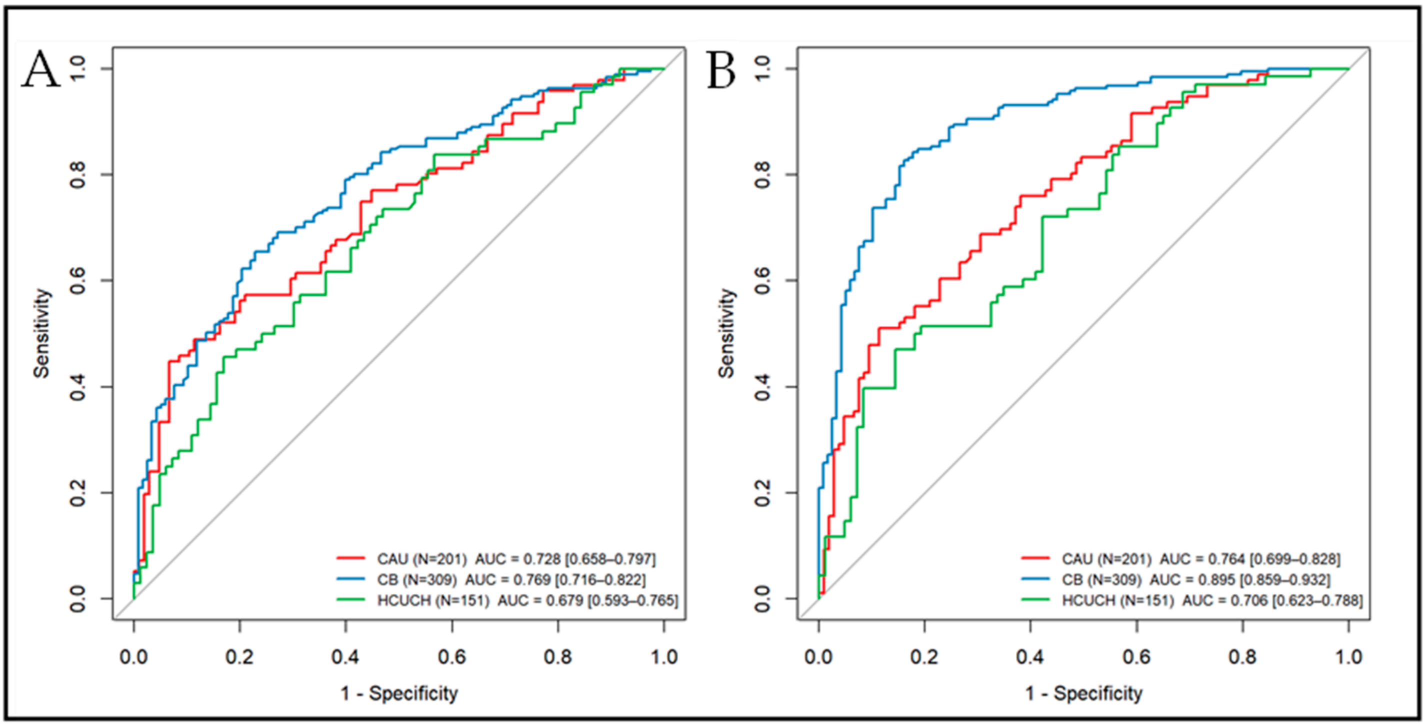

Figure – S4. Discrimination of csPCa (ROC and AUC [95% CI]) at each participant center of BCN-PM 1 (A), and BCN-PM 2 (B).

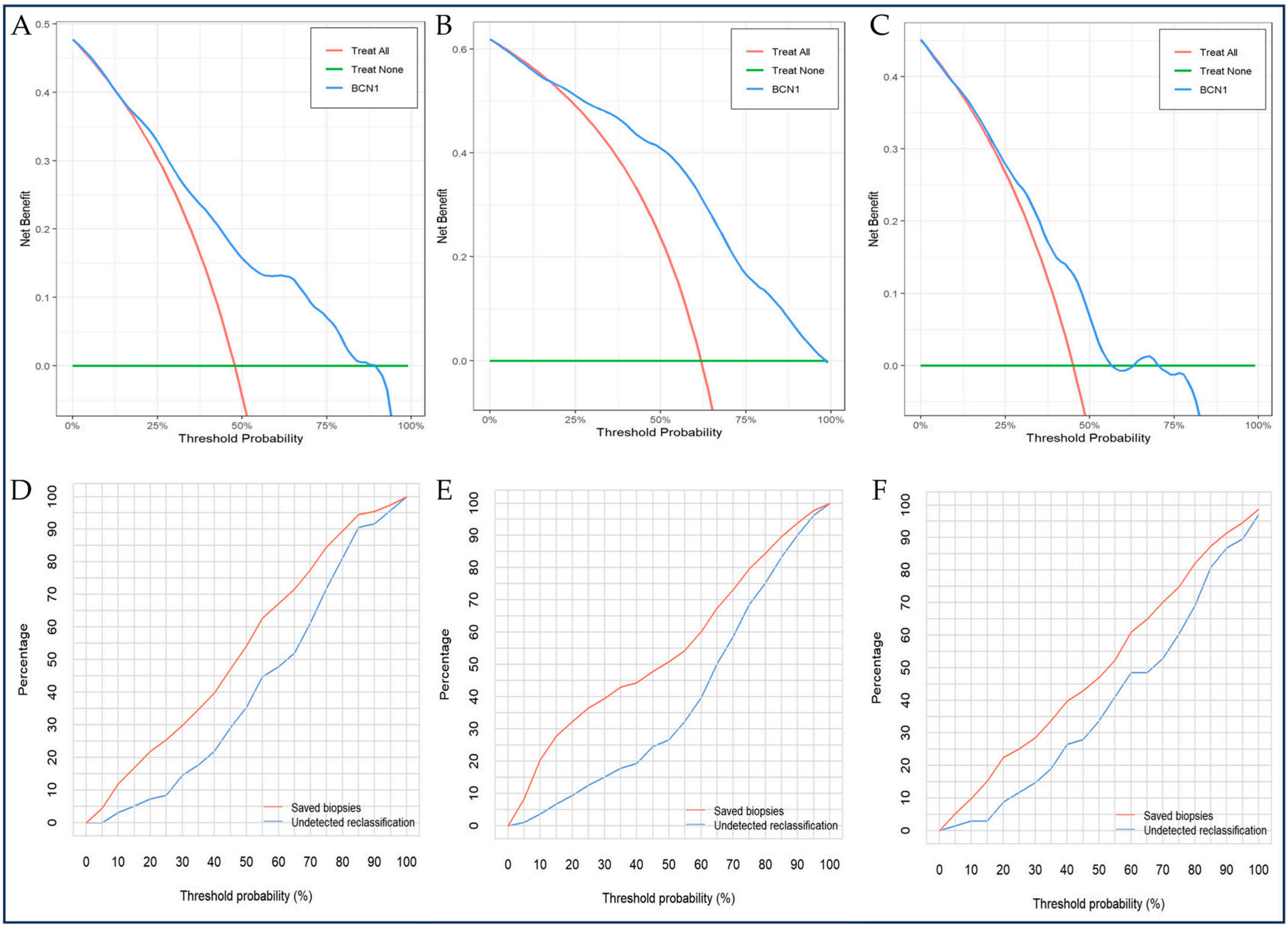

Figure S5. Net benefit (DCA) and clinical utility (CUC) of BCN-PM 1 according to the participant centers. CAU (A and D), CB (B and E), and HCUCH (C and F).

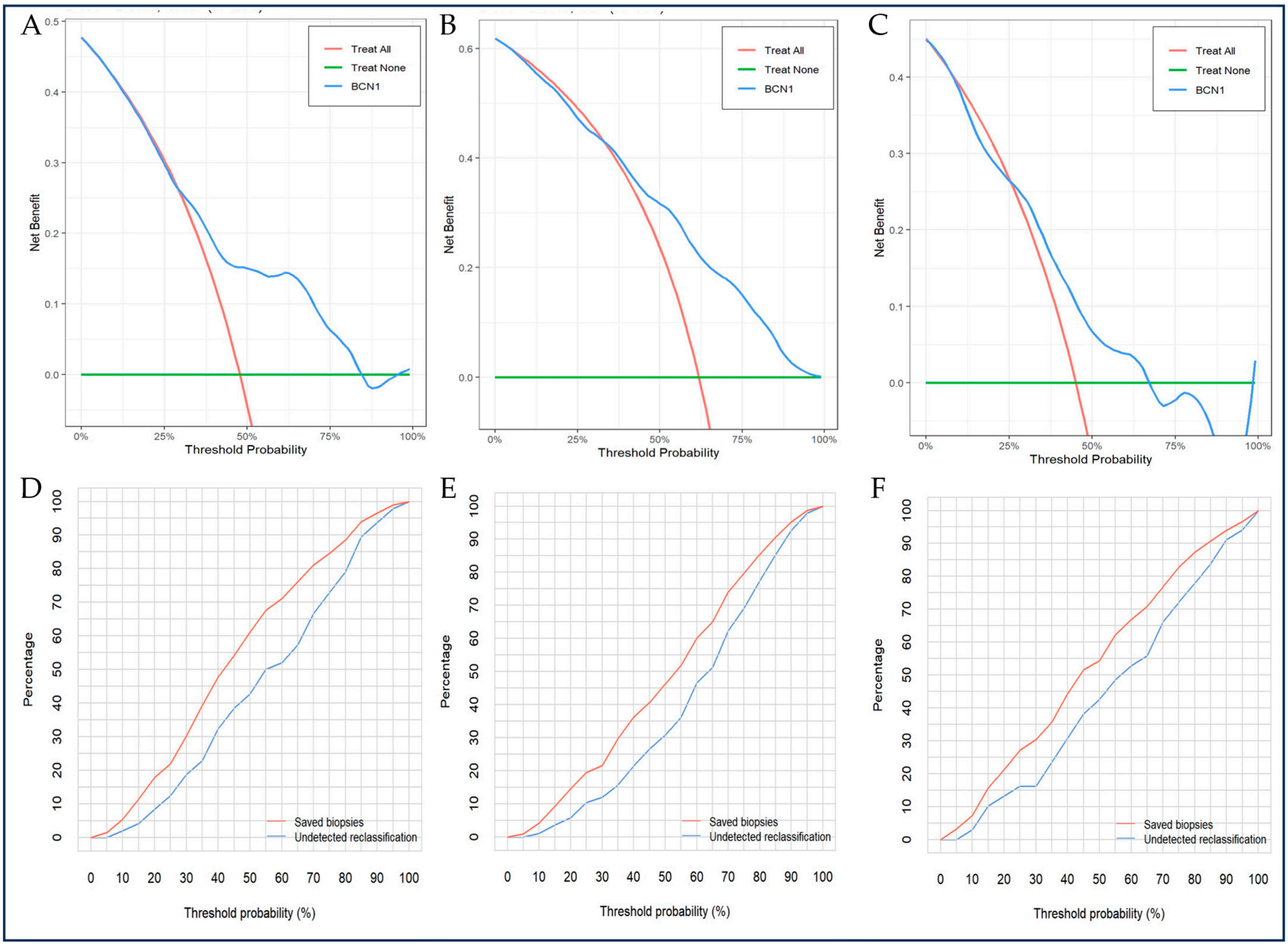

Figure S6. Net benefit (DCA) and clinical utility (CUC) of BCN-PM 2 according to the participant centers. CAU (A and D), CB (B and E-F), and HCUCH (C and F).
